# Reduced circulating FABP2 in patients with moderate to severe COVID-19 may indicate enterocyte functional change rather than cell death

**DOI:** 10.1038/s41598-022-23282-x

**Published:** 2022-11-05

**Authors:** G. Assante, A. Tourna, R. Carpani, F. Ferrari, D. Prati, F. Peyvandi, F. Blasi, A. Bandera, A. Le Guennec, S. Chokshi, V. C. Patel, I. J. Cox, L. Valenti, N. A. Youngson

**Affiliations:** 1 The Roger Williams Institute of Hepatology, Foundation for Liver Research, London, UK; 2 Faculty of Life Sciences & Medicine, King’s College, London, UK; 3 Fondazione IRCSS Ca’ Granda Ospedale Maggiore Policlinico, Milano, Italy; 4 Department of Pathophysiology and Transplantation, Università Degli Studi Di Milano, Milan, Italy; 5 Randall Centre for Cell & Molecular Biophysics, King’s College, London, UK; 6 Institute of Liver Studies, King’s College Hospital, London, UK

**Keywords:** Mechanisms of disease, Apoptosis, Dysbiosis

## Abstract

The gut is of importance in the pathology of COVID-19 both as a route of infection, and gut dysfunction influencing the severity of disease. Systemic changes caused by SARS-CoV-2 gut infection include alterations in circulating levels of metabolites, nutrients and microbial products which alter immune and inflammatory responses. Circulating plasma markers for gut inflammation and damage such as zonulin, lipopolysaccharide and β-glycan increase in plasma along with severity of disease. However, Intestinal Fatty Acid Binding Protein / Fatty Acid Binding Protein 2 (I-FABP/FABP2), a widely used biomarker for gut cell death, has paradoxically been shown to be reduced in moderate to severe COVID-19. We also found this pattern in a pilot cohort of mild (*n* = 18) and moderately severe (*n* = 19) COVID-19 patients in Milan from March to June 2020. These patients were part of the first phase of COVID-19 in Europe and were therefore all unvaccinated. After exclusion of outliers, patients with more severe vs milder disease showed reduced FABP2 levels (median [IQR]) (124 [368] vs. 274 [558] pg/mL, *P* < 0.01). A reduction in NMR measured plasma relative lipid-CH3 levels approached significance (median [IQR]) (0.081 [0.011] vs. 0.073 [0.024], *P* = 0.06). Changes in circulating lipid levels are another feature commonly observed in severe COVID-19 and a weak positive correlation was observed in the more severe group between reduced FABP2 and reduced relative lipid-CH3 and lipid-CH2 levels. FABP2 is a key regulator of enterocyte lipid import, a process which is inhibited by gut SARS-CoV-2 infection. We propose that the reduced circulating FABP2 in moderate to severe COVID-19 is a marker of infected enterocyte functional change rather than gut damage, which could also contribute to the development of hypolipidemia in patients with more severe disease.

## Introduction

Very soon after the onset of the COVID-19 pandemic, the gastrointestinal (GI) tract became a research focus, despite the evident respiratory nature of the disease’s pathogenesis and lethality^1^. The evidence for GI involvement initiated with frequent observation of symptoms such as nausea and vomiting, diarrhoea, abdominal pain, and anorexia. A meta-analysis of 38 studies which included more than 8000 COVID-19 patients showed that 15.5% patients had at least one GI symptom, with 7.5% experiencing nausea/vomiting and 11.5% diarrhoea^2^. GI symptoms are even more common in critically ill patients, with a study reporting the rates of feeding intolerance, abdominal distension, vomiting, constipation and diarrhoea being 56%, 67%, 64%, 37% and 28% respectively^3^.

The discovery that the SARS-CoV-2 virus infects the lungs through binding the receptor angiotensin-converting enzyme 2 (ACE2) increased interest in the GI tract as it is known to be present on enterocytes in the gut epithelium^4^. Subsequent studies confirmed the gut infection route in COVID-19^5,6^ and that the gut is a reservoir for viral particles^1^ which can persist even after clearance from the upper respiratory tract^7^.

Direct invasion of enterocytes leading to cellular changes and cell death is one way in which SARS-CoV-2 can damage the GI tract^8^. However, the GI symptoms in COVID-19 are also likely to be generated by other pathophysiological mechanisms^9^. ACE2 mediates intestinal functions such as the renin–angiotensin–aldosterone system by stimulating intracellular signal pathways when bound. SARS-CoV-2 binding to ACE2 can dysregulate this function^10,11^. Furthermore, the gut microbiome is altered in COVID-19 patients which may lead to imbalance of gut homeostasis, inflammation and dysfunction^12,13^. Finally, the high levels of circulating pro-inflammatory mediators, and immune cell infiltration of the gut may perturb GI function and induce pathology^6^. The multi-faceted role of the gut in COVID-19 infection and severity has led to efforts in treating the disease through modulation of microbiota (probiotics^14–16^ and fecal microbial transplantation^17,18^), diet (e.g. fibre content^1^) and gut function itself (e.g. through regulating plasma serotonin which influences colonic peristaltic reflexes and GI transit^19^).

We investigated the role of the gut in affected COVID-19 patients, grouped according to mild and moderately severe disease, who were admitted to Fondazione Ca’ Grande Ospedale Maggiore Policlinico (Lombardy, Italy)^20^. For this pilot study we assayed commonly used plasma biomarkers of gut permeability (zonulin) and enterocyte cell death (Intestinal Fatty Acid Binding Protein / Fatty Acid Binding Protein 2, I-FABP/FABP2) as well as a range of plasma metabolites with nuclear magnetic resonance (NMR) spectroscopy^21^. The earliest available timepoint plasma sample (closest to date of admission) was analysed and compared to patient clinical data recorded within 3 days of the experimental sample collection. We hypothesised that circulating plasma markers of gut inflammation and damage would differ according to severity of disease and could correlate to circulating levels of metabolites as measured by NMR spectroscopy.

## Results

### General characteristics of the pilot study cohort

Patients were grouped based on the ultimate level of breathing support that they received. Patients receiving nasal cannula (*n* = 17) or VentMask (*n* = 1) were placed in the mild group, while those who received CPAP were grouped as moderately severe (*n* = 19) (Table 1). Importantly, we use the classification ‘moderately severe’ as no patients were sedated and on parenteral nutrition receiving mechanical ventilation or extracorporeal membrane oxygenation (ECMO).

Within the moderately severe group, five patients (26.3%) succumbed to death. The gender and age distributions of the two groups were similar, and the only significantly different comorbidity was obesity, which was more common in the moderately severe group, though it was not possible to ascertain in all participants (Supplementary File 1).

Multiple measurements of blood cells and molecules were at significantly different levels between the two groups in line with several studies which have reported COVID-19 associated changes (Table 1 and Supplementary File 1). For example, patients with more severe COVID-19 had elevated neutrophils, C-reactive protein (CRP), procalcitonin (Pct), ferritin and fibrinogen but reduced lymphocytes compared to milder COVID-19 patients.

**Table 1:** Selected clinical and demographic parameters of the study population.

Characteristic	Mild^a^	Moderately**-**Severe^b^	*P* value
No. of subjects	18	19	
Male, n (%)	13 (72.2)	15 (78.9)	
Female, n (%)	5 (27.8)	4 (21.1)	
Age in yrs, median (IQR^c^)	60.6 (52.0 − 64.0)	59.9 (55.1 − 66.2)	0.23
BMI, median (IQR)	26.3 (25.0 − 28.4) [n = 8]	28.5 (26.4 − 37.9) [n = 10]	0.20
AST Liver Enzyme, median (IQR)	31.5 (23.8 − 47.5) [*n* = 10]	52.5 (36.8 − 75.8) [*n* = 10]	0.19
ALT Liver Enzyme, median (IQR)	36.0 (29.3 − 42.0)	40.0 (30.0 − 58.0)	0.70
Lymphocytes, median (IQR)	1.47 (1.17 − 1.94)	0.81 (0.66 − 1.23)	0.002
Neutrophils, median (IQR)	4.13 (2.34 − 5.59)	5.34 (4.46 − 7.52)	0.019
CRP, median (IQR)	2.27 (1.08 − 4.37)	13.35 (8.22 − 24.0)	0.0003
Pct, median (IQR)	0.09 (0.05 − 0.14)	0.33 (0.15 − 1.14)	0.032
Ferritin, median (IQR)	333 (223 − 407)	932 (568 − 1635)	0.014
Fibrinogen, median (IQR)	439 (405 − 646)	625 (506 − 737)	0.015

*CRP* C reactive protein; *AST* aspartate aminotransferase; *ALT* Alanine aminotransferase.

^a^Nasal cannula or VentMask, ^b^CPAP, Continuous positive air pressure, ^c^IQR, interquartile range.

### Gut damage marker ELISAs

Plasma zonulin levels and FABP2 levels were measured in 17 milder COVID-19 patients and 16 more severe COVID-19 patients.

**Figure 1:**
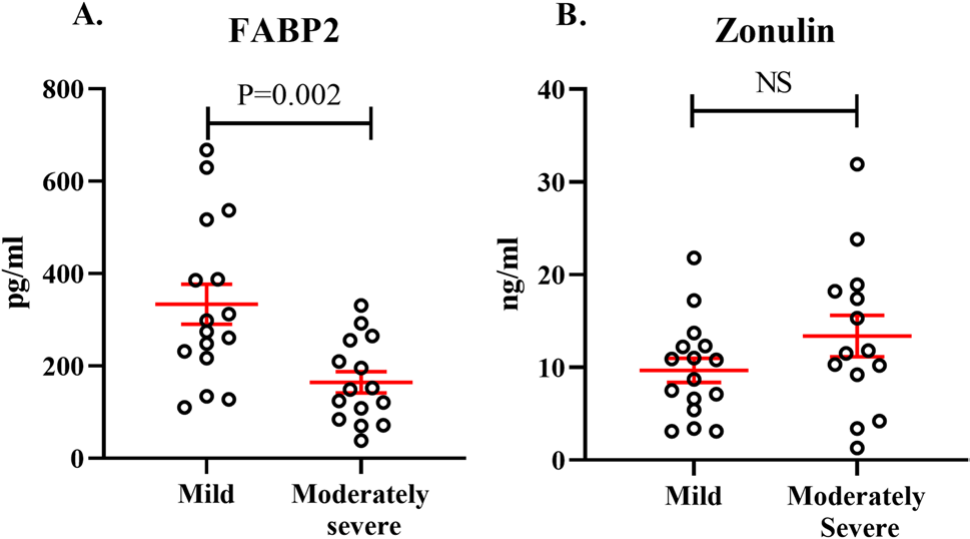
Plasma FABP2 but not zonulin is decreased in patients with more severe COVID-19 compared to patients with milder COVID-19. (**A**) Plasma FABP2 levels in patients with milder (*n* = 16) or more severe COVID-19 (*n* = 15), respectively. (**B**) Plasma zonulin levels in patients with milder (*n* = 16) or more severe (*n* = 14) COVID-19, respectively. Student’s t-test used for group comparisons, mean ± SEM indicated with red lines.

For the zonulin ELISA measurements, one and two samples were respectively excluded from the mild [ID 503, zonulin 76.3 ng/ml] and moderately severe groups [ID 506, 562, zonulin 48.4 ng/mL, 47.5 ng/mL respectively] for being more than two standard deviations from the mild and moderately severe group means (9.7 and 13.4 ng/mL respectively). Zonulin was slightly higher (not significantly, *P* = 0.15) in the more severe group (*N* = 14) than the milder group (*N* = 16) (Fig. 1). When the outliers were not excluded the *P* value was 0.46.

For the FABP2 ELISA, one sample from the milder group [ID 291, FABP2 1415.9 pg/mL] and one from the more severe group [ID 290, FABP2 406.2 pg/mL] were excluded for being more than two standard deviations from the group means (333.7 and 164.5 pg/mL respectively. FABP2 levels were significantly reduced (*P* = 0.002) in the more severe group (*N* = 15) compared to the mild group (*N* = 16) (Fig. 1). When the outliers were not excluded the group difference was still statistically significant *P* = 0.01.

### Plasma NMR

**Figure 2:**
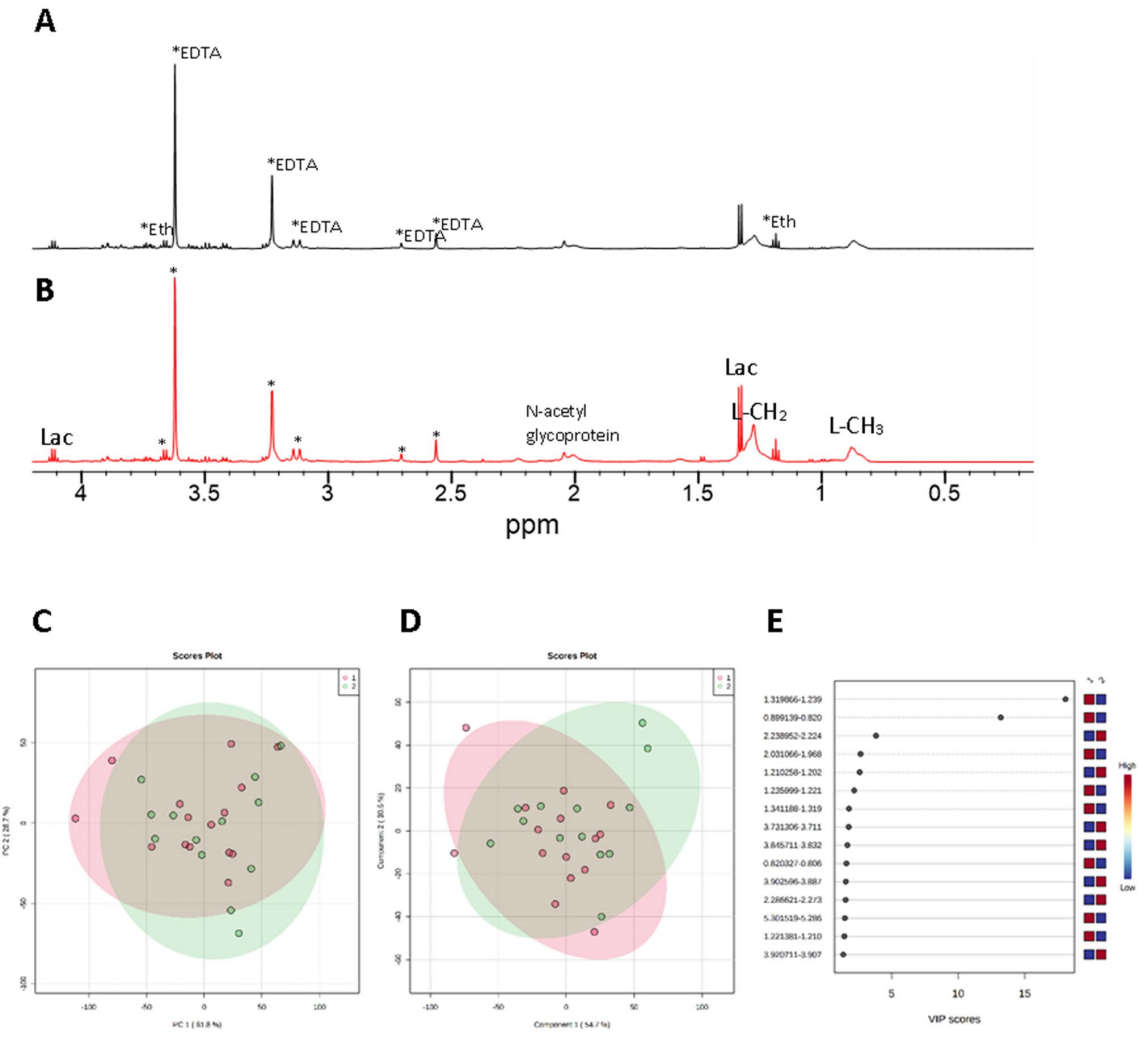
(**A**,**B**) Illustrative expanded NMR spectra from patients in the moderately severe Covid group with differing FABP2 levels. (**A**) ID 112, FABP2 38 pg/mL, (**B**) ID 433**,** FABP2 195 pg/mL. The peaks marked with * were excluded from the analysis and include EDTA peaks (3.635–3.600 ppm; 3.235–3.220 ppm, 3.18–3.07 ppm; 2.72–2.68 ppm; 2.58–2.53 ppm) and ethanol peaks (quartet 3.685–3.635 ppm, triplet 1.20–1.06 ppm). Lac, lactate; L–CH_2_, lipid-CH_2_ peak; L–CH_3_, lipid-CH_3_ peak. (**C**) Principal Component Analysis showing no difference between the mild [1, pink] and moderately severe [2, green] groups. (**D**) Partial Least Squares Discriminant Analysis showing no difference between the mild [1, pink] and moderately severe [2, green] groups. (**E**) Variable in Importance Projection plot showing L–CH_2_ and L–CH_3_ to be the top discriminating metabolites between the mild [1] and moderately severe [2] groups.

Thirty-two EDTA plasma samples were available for NMR analysis (milder COVID-19 group *N* = 17, more severe COVID-19 group *N* = 15). Illustrative NMR spectra from the more severe COVID-19 group are shown in Fig. 2, noting that the NMR spectra are dominated by peaks from the sampling contaminants EDTA and ethanol.

There was no difference between the NMR spectra from milder and more severe COVID-19 groups using principal component analysis (PCA) and partial least squares discriminant analysis (PLS-DA), which was confirmed by no difference between groups on cross validation (Q2 < 0). The Variable in Importance Projection plots showed lipid-CH_3_ and lipid-CH_2_ to be the most discriminatory metabolite regions. The medians and IQRs for selected metabolites in the mild and moderately severe patient groups are presented in Supplementary Table 1. Only lipid CH_3_ approached significance (*P* = 0.06) (t-test), between groups, being reduced in the moderately severe group compared to the mild group.

### NMR associations within Covid severity groups

Relative NMR metabolite levels from 32 patients (milder COVID-19 group *N* = 17, more severe COVID-19 group *N* = 15) were compared to FABP2 and zonulin concentrations using Pearson r correlation (GraphPad Prism 9.2.0). Four data points (*n* = 2 mild COVID-19, ID 503, 291; *n* = 2 moderately severe COVID-19 group, ID 506, 562; refer to ELISA exclusions above) were identified as outliers and so these data points were excluded from further analysis.

**Figure 3:**
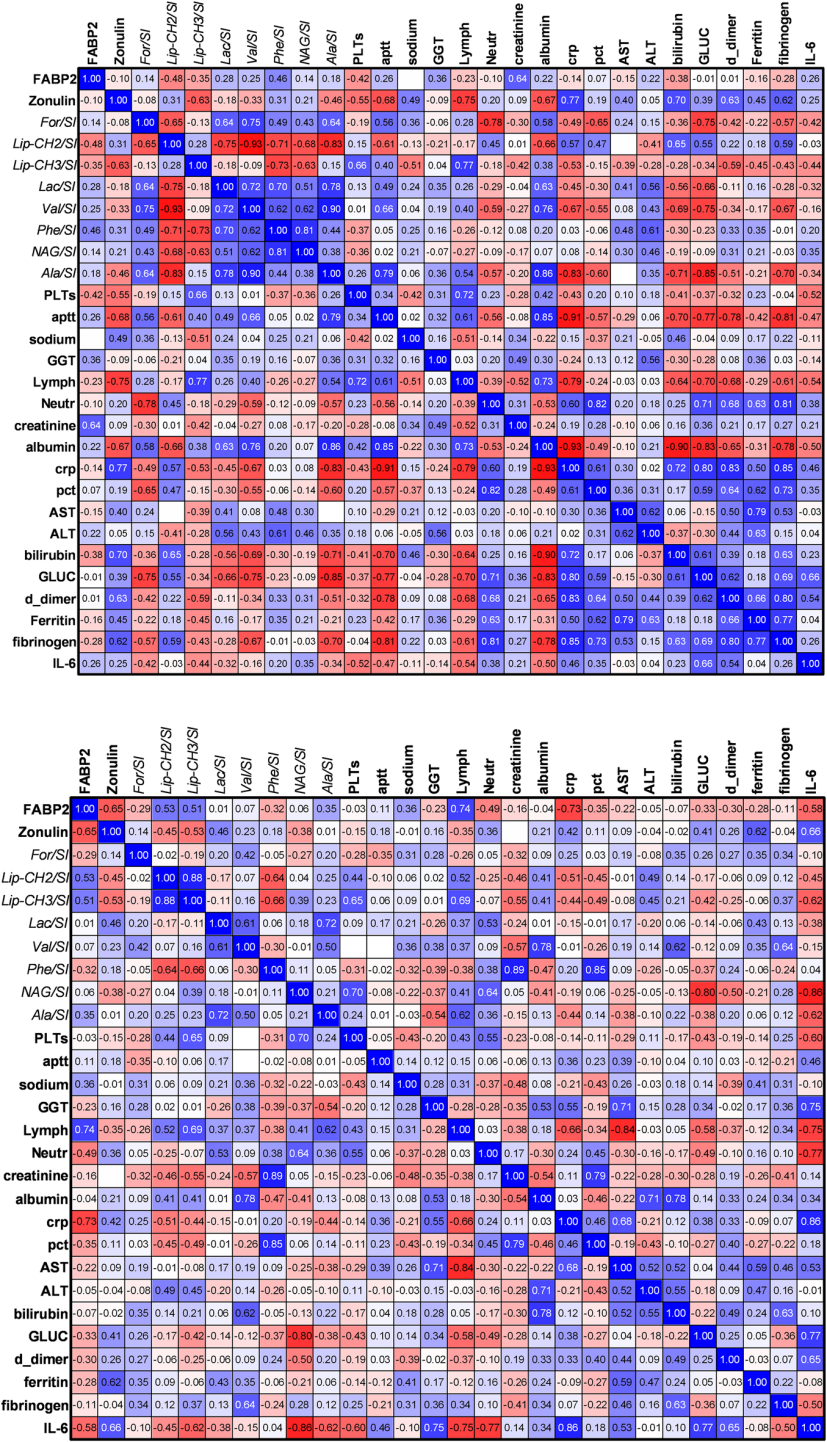
(**A**) Correlation Pearson R matrix showing association between gut biomarkers and plasma NMR metabolites for the milder group (*N* = 15), after two outliers were removed, as determined by ROUT (ID 503, 291). (**B**) Associations between gut biomarkers and plasma NMR metabolites for the more severe group (*N* = 13), after two outliers were removed, as determined by ROUT analysis (ID 506, 562). Blue indicates a positive association, red indicates a negative association. Stronger associations indicated by deeper colour.

Correlation plots for the mild COVID-19 group (*N* = 15) and moderately severe COVID-19 group (*N* = 13) are shown in Fig. 3A,B respectively. The strongest positive associations in the more severe COVID-19 group, (i.e. low level when FABP2 is low) were in plasma lipids (both lipid-CH_2_ and lipid-CH_3_ regions) and lymphocytes.

## Discussion

From onset of the COVID-19 pandemic the gut has repeatedly been implicated in infection, progression and severity of the disease. We and others considered the role of microbial translocation in exacerbating the hyperinflammation in moderate to severe COVID-19^22,23^. There is now strong evidence for increasing microbial translocation in COVID-19 which correlates with inflammation^24–26^ and may even influence mortality^27^. Mechanistically, it is unclear whether the increased translocation is due to gut damage caused by enterocyte infection^5,6,28^, or whether SARS-CoV-2 infected lung induces systemic hyperinflammation, microbial dysbiosis, and/or coagulopathy which in turn causes the gut damage^13,29,30^. One way to answer this question is to compare the levels of plasma biomarkers of gut permeability and damage with markers of inflammatory responses across the different disease stages. Gut permeability markers such as LPS, LBP, zonulin and cCD14 have been shown to be higher in COVID-19 patients than healthy controls^24,25,31,32^. Giron et al., (2021) examined gut-associated biomarkers and metabolites in a cohort of patients with mild (outpatients), moderate (inpatients hospitalised in regular wards) and severe (inpatients hospitalised in ICU) COVID-19 (18–20 in each group)^24^. They observed large and significant increases in the tight junction permeability marker zonulin between the mild and moderate/severe groups, but no difference between the latter two. Therefore, in this cohort at least the biggest increases in gut permeability occur between the timepoints of infection and hospitalisation, with little further increase in the more severe stages which are associated with hyperinflammation. Our study of relatively mild and more severe COVID-19 patients (as determined by the level of respiratory support) admitted in medicine units, also showed little increase in zonulin between these stages.

As gut permeability and enterocyte cell death go hand in hand in conditions such as sepsis and intestinal ischaemia/reperfusion^33^, and as SARS-CoV-2 infection has been observed to kill gut epithelial cells^8^, we expected that plasma FABP2 would also increase in COVID-19 patients. However, we saw the opposite, with a significant reduction in more severe compared to milder COVID-19 patients. The literature on plasma FABP2 in COVID-19 is conflicted, with some studies reporting increased FABP2 in COVID-19 patients compared to healthy controls^25,32^, some no difference^24,31,34^ and some a reduction^35,36^. Studies which classified disease condition as mild (outpatients), moderate (inpatients hospitalised in regular wards) and severe (hospitalisation in ICU) have either shown no increases^34,35^ or a similar decrease in severe compared to moderate as we saw^25,36^.

Further studies are therefore needed to confirm the relationship between plasma FABP2 levels and COVID-19 severity. However, the surprising decrease in the more severe patients deserves consideration as it gives insight into the state of the gut at the most critical timepoint for patient survival. A straightforward explanation could be that the expression of FABP2 is reduced in these patients due to reduced food intake or malabsorption^3,37,38^. However, since in the present study we did not examine the most severe patients who were on mechanical ventilation, and therefore sedated and on parenteral nutrition, we ruled out a possible source of major bias in previous reports. Alternatively, or additionally, plasma FABP2 levels may be reduced as the gene’s expression is downregulated in enterocytes due to infection or an aspect of hyperinflammation itself. In vitro experiments with human small intestinal organoids found that fat metabolism and biosynthesis were some of the most significantly downregulated processes in response to SARS-CoV-2 infection^5^ (FABP2 itself was reduced but non-significantly). This metabolic change has been proposed to be an anti-viral measure, as SARS-CoV-2 infection and replication makes extensive use of cellular lipids^39,40^. This alteration of gut lipid metabolism may even have importance for disease survival as the drug Ezetimibe which reduces fat uptake in the gut^41^ has been reported to possibly reduce COVID-19 severity^42^.

Comparison of plasma FABP2 levels with other circulating metabolites and patient clinical data in the more severe COVID-19 group showed the strongest negative associations (i.e. high level when FABP2 is low) with CRP, Il–6 and zonulin. These data suggest that plasma FABP2 is especially low in patients with the highest levels of inflammation and gut permeability. The anti-correlation between plasma FABP2 and systemic inflammation fits with the observation that the 3 lowest FABP2 levels in the study were recorded in patients who died (out of a total of 5 who died). However, the significant difference in FABP2 levels between the 2 study groups was still seen if these patients were excluded. The strongest positive associations in the more severe COVID-19 group, (i.e. low level when FABP2 is low) were in plasma lipids (both lipid intramolecular CH_2_ and CH_3_ groups), and lymphocytes, both of which have been frequently observed in severe COVID-19^40,43,44^. However, considering the low sample numbers of these comparisons, care must be taken in their interpretation.

Several studies have focussed on the plasma lipid changes in COVID-19 patients^39,40,45–50^. It is important to note that patients with type 2 diabetes, obesity, or users of lipid lowering drugs are more likely to progress to severe COVID-19 due to their comorbidities, so this needs to be taken into account when stratifying patients into severity groups in order to investigate lipids in COVID-19^51^. Accordingly, low HDL cholesterol and high triglycerides prior to infection are risk factors for progression to severe COVID-19^46^. Nonetheless, the most commonly reported lipid changes in COVID-19 patients at all stages of disease are reduced circulating cholesterol (LDL and HDL) and elevated triglycerides^21,47–50,52^. Reported metabolite changes includes increased levels of acetoacetic acid, 3-hyroxybutyric acid, acetone and 2-hdroxybutyic acid; changes have also been reported in porphyrin levels, branched chain amino acids and tryptophan pathways^21,45,49,53^. The most severely affected patients have a further reduction of HDL compared to patients with moderate disease^46,47^, with increases of cholesterol and triglycerides upon recovery in survivors^52^. As FABP2 expression level is an important determinant of lipoprotein production in the gut it is possible that it may contribute to the reduction in HDL in the more severely affected patients^54–59^.

The extreme and progressive immunological response induced by SARS-CoV-2 infection explains many of the differences in systemic inflammatory markers between the milder and more severe groups^43^. Higher neutrophil to lymphocyte ratio, CRP and procalcitonin have all been used to stratify severity of COVID-19^44^. Elevated ferritin has also been reported in many COVID-19 studies^60^. Ferritin correlates with pulmonary involvement in SARS-CoV-2 infection^61^, which fits with our use of pulmonary support to separate the two patient groups. Higher levels of the clotting factor fibrinogen are also associated with severe COVID-19^62^. Overall, these data indicate that there is a higher systemic inflammation response, particularly of the acute phase reaction in the more severe patient group than the mild group.

Limitations to our study were linked to the low number of patients examined and the low volume of plasma available. The period of sample collection was during a particularly challenging time in Milan, since it was the first major European city to experience the COVID-19 pandemic. Consequently, patient samples for highly selected research purposes were scarce ^63,64^. The low sample volume available meant we had to adjust the routine replicate experimental practices into singlicate analysis and use a minimal volume of 70 μl of plasma for NMR. A more extensive study of circulating markers of gut function, such as citrulline and gut cell death, is needed to confirm that the FABP2 levels in severe COVID-19 are indicative of altered lipid import and metabolism rather than apoptosis. Additionally, sample collection used EDTA as an anticoagulant, so a number of NMR peaks were obscured by the EDTA resonances, including those that would have given information on choline-containing compounds.

Another consequence of low sample numbers is that, unlike some published studies with larger patient groups, the NMR metabolites were not significantly different between the mild and moderately severe groups, with only lipid CH_3_ approaching significance with a reduction in the moderately severe group (*P* = 0.06) compared to the mild group. Plasma NMR spectroscopy is high in information content^65^. Therefore, detailed interpretation of plasma NMR findings may require information from a range of clinical and lifestyle factors, to allow for the impact of confounding factors on metabolite levels. It is therefore possible that increased sample numbers would have enabled subgroups to be analysed to fully account for confounding variables, such as gender, co-morbidities, medication and nutritional status. The presence of correlations between the ELISA measured parameters and NMR datasets seems to confirm that possibility. As previously discussed, there is an abundance of literature on reductions in plasma lipid species in COVID-19 patients^21^, and our observed trends of formate^53,66^, phenylalanine^21,66–69^ and 3-hydroxybutyrate^21,49,66–69^ increasing with COVID-19 severity is in agreement with previous plasma NMR studies^21^.

In conclusion our data strengthen the possibility that downregulation of enterocyte fat metabolism in patients with moderate to severe COVID-19 is contributing to the reduced circulating cholesterol, potentially as an anti-infection response. As has been proposed by others, our data therefore support the idea that manipulation of gut lipid absorption is a useful therapeutic approach to manage COVID-19^42,70^.

## Methods

### Clinical parameters

All experiments were performed in accordance with the ethics committee guidelines and regulations (COVID-19 and non-COVID patients). Informed consent was obtained from all participants and/or their legal guardians (COVID-19 and non-COVID patients).

Ethics was obtained for the “Fondazione Genomic SARS-CoV-2 study” (ethics number 109365) to Fondazione IRCCS Ca’ Granda Ospedale Maggiore Policlinico Milano. Blood samples were obtained with informed consent and plasma samples were collected into EDTA tubes. All studies were performed in accordance with guidelines and regulations. The clinical metadata was compiled by the Biological Resource Center and Translational Medicine Unit, Dipartimento di Medicina Trasfusionale e di Ematologia, Fondazione IRCCS Ca' Granda Ospedale Maggiore Policlinico, Università degli Studi di Milano.

Non-COVID patient samples for assay optimisation were recruited at King’s College Hospital after admission to the ward or from the hepatology out-patient clinic. The study was granted ethics approval by the UK NHS national research ethics committee (Apprival number 12/LO/1417) and local research and development department at King’s College Hospital (Approval number KCH12-126).

### Laboratory measurements

Laboratory measurements were made in 33 plasma samples from COVID-19 patients in a CL2 laboratory housed at the Institute of Liver Studies, Kings College Hospital. All assays were performed using plasma which was not treated to inactivate the virus (e.g. heat-inactivation) so as to avoid denaturation of proteins which can impair antibody detection.

To ensure safety while handling samples which potentially contained live SARS-CoV-2 several steps and procedures were undertaken. Tubes containing patient plasma were aliquoted within a biosafety hood in the CL3 laboratory (at the Roger Williams Institute of Hepatology). The initial steps of ELISAs were performed in the Mowat Laboratories biosafety hoods for blood handling with 96-well plates only removed from the hoods after the post-primary antibody incubation wash stage.

Active (uncleaved) zonulin was measured using a sandwich enzyme immunoassay (Human Zonulin ELISA Kit MyBioSource MBS706368) with manufacturer’s instructions.

Plasma Intestinal fatty acid-binding protein (I-FABP/FABP2) were measured using a sandwich enzyme immunoassay (Human FABP2/I-FABP Quantikine ELISA Kit, R&D systems DFBP20). The manufacturer’s instructions were followed with plasma diluted fivefold in sample diluent (22 μl plasma added to 88 μl diluent). Prior to analysis with COVID-19 samples we investigated whether heat-inactivation of the plasma, a commonly used method to destroy SARS-CoV-2 virus, could influence FABP2 assay fidelity. We performed the ELISA using plasma samples from four patients with decompensated liver disease and one healthy control. An aliquot of each sample underwent heat inactivation of the SARS-CoV-2 virus with incubation at 56 °C for 15 min. As expected without heat inactivation the healthy control sample (298.1 pg/mL) was lower than the samples from patients with decompensated liver cirrhosis (mean 789.3 ± 182.1 pg/mL SEM)^71^. However, heat inactivation drastically reduced the levels detected in all samples, even to below the level of detection in two samples, presumably by denaturing FABP2 protein and destroying the immunological detection site. We therefore examined FABP2 and zonulin in the COVID-19 samples without heat inactivation.

In the analysis of COVID-19 patient samples both zonulin and FABP2 were measured only once from the samples due to limitations of available sample volume. End-point analysis for both ELISAs was performed using a FLUOstar Omega microplate reader.

### Plasma NMR studies

EDTA plasma samples were collected in sufficient volume for NMR study from 32 patients. Samples were stored frozen at − 80 °C until NMR analysis at the Centre for Biomolecular Spectroscopy, King’s College London. NMR results from the earliest time point (day 1 for all *N* = 32 subjects) have been included.

On the day of NMR analysis, the EDTA plasma samples were gently thawed. 70 µl of plasma and 110 µl of 75 mM sodium phosphate buffer solution (with 6.2 mM sodium azide and adjusted to pH 7.4) were aliquoted into 3 mm SampleJet NMR tubes (Bruker BioSpin, Germany). NMR data were also acquired from three samples of fetal calf serum to illustrate and confirm stability and reproducibility of the NMR spectrometer. The samples were transported at 4 °C locally to the Centre for Biomolecular Spectroscopy at Guy’s Campus, King’s College London.

On delivery to the NMR facility, the SampleJet racks were immediately placed on the SampleJet holder for sample storage at 4 °C prior to data collection. Proton (1H) NMR spectra were acquired at 37 °C (310 K) using a Bruker 600 MHz (AVANCE NEO) NMR spectrometer and a 1H/13C/15 N TCI Prodigy probe (nitrogen-cooled). Proton shimming was done under automation such that a 1.0–1.5 Hz linewidth for one of the alanine doublet peaks was routinely achieved. Pulse-collect and spin-echo 1D NMR data sets were acquired using PURGE water suppression and the PROJECT spin-echo sequences, as previously described^72^. Both data sets were acquired with 4 dummy scans, 64 data collects, constant receiver gain, 64 K points, acquisition time 2.62 s and recycle delay of 4 s. The spin-echo time for the PROJECT sequence is 78 ms (64 loops). For confirmation of peak assignment, a TOCSY spectrum and an HSQC spectrum was acquired on a representative plasma sample.

The 1D NMR data sets were processed using 0.3 Hz exponential line broadening filter. Metabolite assignments were made based on chemical shift and coupling patterns with reference to published databases and confirmed by the TOCSY and HSQC NMR studies^73^.

Mulitvariate analyses of the NMR spectral region 10.00–0.50 ppm were undertaken using spectral binning methodologies as previously published^74,75^. Specifically, KnowItAll software (Wiley Science Solutions KnowItAll Spectroscopy Edition software version 17.0.117.0 (https://sciencesolutions.wiley.com/knowitall-spectroscopy-software/) and MetaboAnalyst v5.0 (https://www.metaboanalyst.ca) were used. Certain regions of all NMR data sets were excluded from multivariate analyses because of confounding effects, which included the residual water region (5.00–4.50 ppm), EDTA peaks (3.635–3.600 ppm; 3.235–3.220 ppm, 3.18–3.07 ppm; 2.72–2.68 ppm; 2.58–2.53 ppm) and ethanol contamination (quartet 3.685–3.635 ppm, triplet 1.20–1.06 ppm). Both fixed width spectral bins (0.02 ppm bucket widths) and variable width spectral bins (defined using the proprietary InetillibucketTM software of Wiley Sciences Solutions KnowItAll, which is based on defining local minima) were used to define metabolite regions. Following on from variable width spectral bucketing, specific regions were targeted to minimise any peak overlap, including formate, 8.468–8.455 ppm; phenylalanine, 7.452–7.414 ppm; N-acetyl glycoproteins, 2.05–2.03 ppm; alanine, 1.495–1.470 ppm; lactate, 1.34–1.32 ppm; lipid CH_2_, 1.32–1.24 ppm; lipid CH_3_, 0.90–0.82 ppm; valine, 1.057–1.023 ppm. Peaks were also quantified for acetone (2.06–2.03 ppm) and 3-hydroxybutyrate (region not overlapping with ethanol triplet, 1.21–1.20 ppm) and assigned to leucine and isoleucine.

### Statistics

Differences for specific parameters between clinical groupings were assessed using Student’s t-tests (IBM SPSS Statistics v28.0.1.1).

MetaboAnalyst v15 (https://www.metaboanalyst.ca) was used to compare NMR changes between clinical groupings using principal component analysis (PCA) and partial least squares discriminant analysis (PLS-DA).

Alterations in relative levels of circulating metabolites were correlated with measures of gut permeability (FABP2 and zonulin) using Pearson r Correlation (GraphPad Prism 9.2.0, Graphstats Technologies Private Limited, Karnataka, India). Outliers were determined by the ROUT algorithm in GraphPad Prism.

## Supplementary Information

Supplementary Information 1.

Supplementary Information 2.

## Data Availability

The datasets generated during and/or analysed during the current study are available from the corresponding author on reasonable request.
