# Supplementary material for: Reduced circulating FABP2 in patients with moderate to severe COVID-19 may indicate enterocyte functional change rather than cell death
Source: Sci Rep. 2022 Nov 5;12:18792. doi: 10.1038/s41598-022-23282-x (PMC9637119; doi:10.1038/s41598-022-23282-x)
Supplement: Supplementary file 1 — Supplementary Information 1. [file 41598_2022_23282_MOESM1_ESM.pdf]

SUPPLEMENTARY TABLE 1. NMR metabolite group comparison

**Characteristic**	**Mild** [a]	**Moderately-Severe** [b]	**P value**
No. of subjects	17	15	
3-hydroxybutyrate / SI[c] median (IQR[d])	0.0038 (0.0036-0.0045)	0.0041 (0.0035-0.0046)	0.31
Acetone /SI median (IQR)	0.0259 (0.0241-0.0273)	0.02461 (0.0235-0.0.0271)	0.55
Alanine /SI median median (IQR)	0.0064 (0.0054-0.0073)	0.0055 (0.0045-0.0063)	0.13
Formate / SI median (IQR)	0.000125 (0.00009-0.00014)	0.000131 (0.00009-0.00017)	0.52
Lactate /SI median (IQR)	0.0750 (0.0624-0.0853)	0.0739 (0.0617-0.0869)	0.91
Lipid CH2 /SI median (IQR)	0.1609 (0.1387-0.1792)	0.1499 (0.1267-0.1897)	0.39
Lipid CH3 /SI median (IQR)	0.0811 (0.0775-0.0886)	0.0729 (0.0628-0.0870)	0.06
N-acetyl glycoproteins /SI median (IQR)	0.0259 (0.0241-0.0273)	0.0246 (0.0235-0.0271)	0.55
Phenylalanine /SI median (IQR)	0.00061 (0.0005-0.0007)	0.00065 (0.0005-0.0008)	0.48
Valine /SI median (IQR)	0.0038 (0.0032-0.0045)	0.0034 (0.0032-0.0039)	0.26

[a] Nasal cannula or VentMask

[b] CPAP, Continuous Positive Air Pressure

[c] SI. Signal Intensity summed from the analysed regions of each NMR spectrum

[d] IQR, interquartile range.
